# Supplementary material for: Effect of Interferon Gamma on Ebola Virus Infection of Primary Kupffer Cells and a Kupffer Cell Line
Source: Viruses. 2023 Oct 11;15(10):2077. doi: 10.3390/v15102077 (PMC10611415; doi:10.3390/v15102077)
Supplement: Supplementary file 1 [file viruses-15-02077-s001.zip › viruses-2605884-supplementary.pptx]

## Slide 1
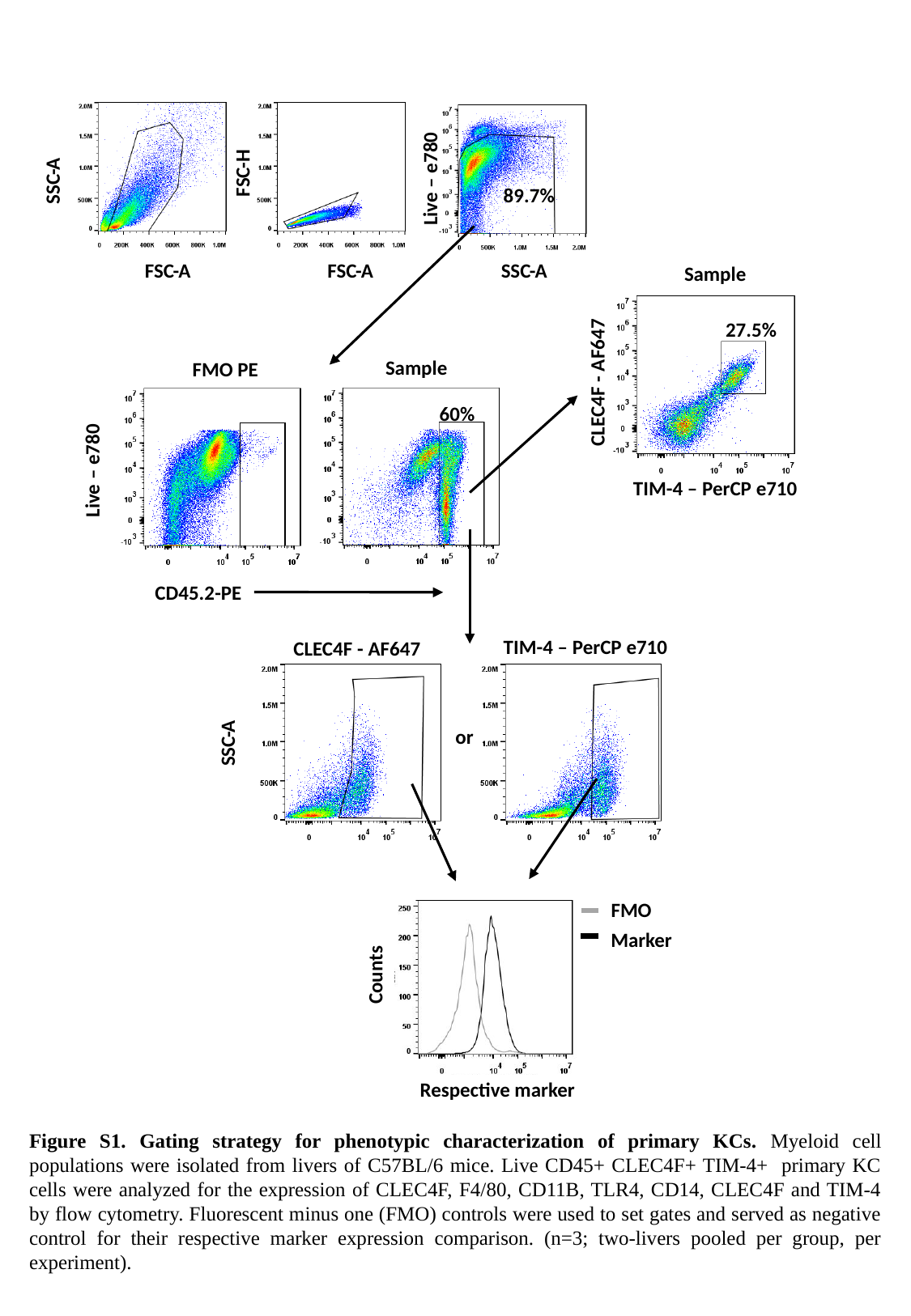

FSC-H
Live – e780
SSC-A
89.7%
FSC-A
FSC-A
SSC-A
Sample
27.5%
Sample
FMO PE
CLEC4F - AF647
60%
Live – e780
TIM-4 – PerCP e710
CD45.2-PE
TIM-4 – PerCP e710
CLEC4F - AF647
or
SSC-A
FMO
Marker
Counts
Respective marker
Figure S1. Gating strategy for phenotypic characterization of primary KCs. Myeloid cell populations were isolated from livers of C57BL/6 mice. Live CD45+ CLEC4F+ TIM-4+ primary KC cells were analyzed for the expression of CLEC4F, F4/80, CD11B, TLR4, CD14, CLEC4F and TIM-4 by flow cytometry. Fluorescent minus one (FMO) controls were used to set gates and served as negative control for their respective marker expression comparison. (n=3; two-livers pooled per group, per experiment).

## Slide 2
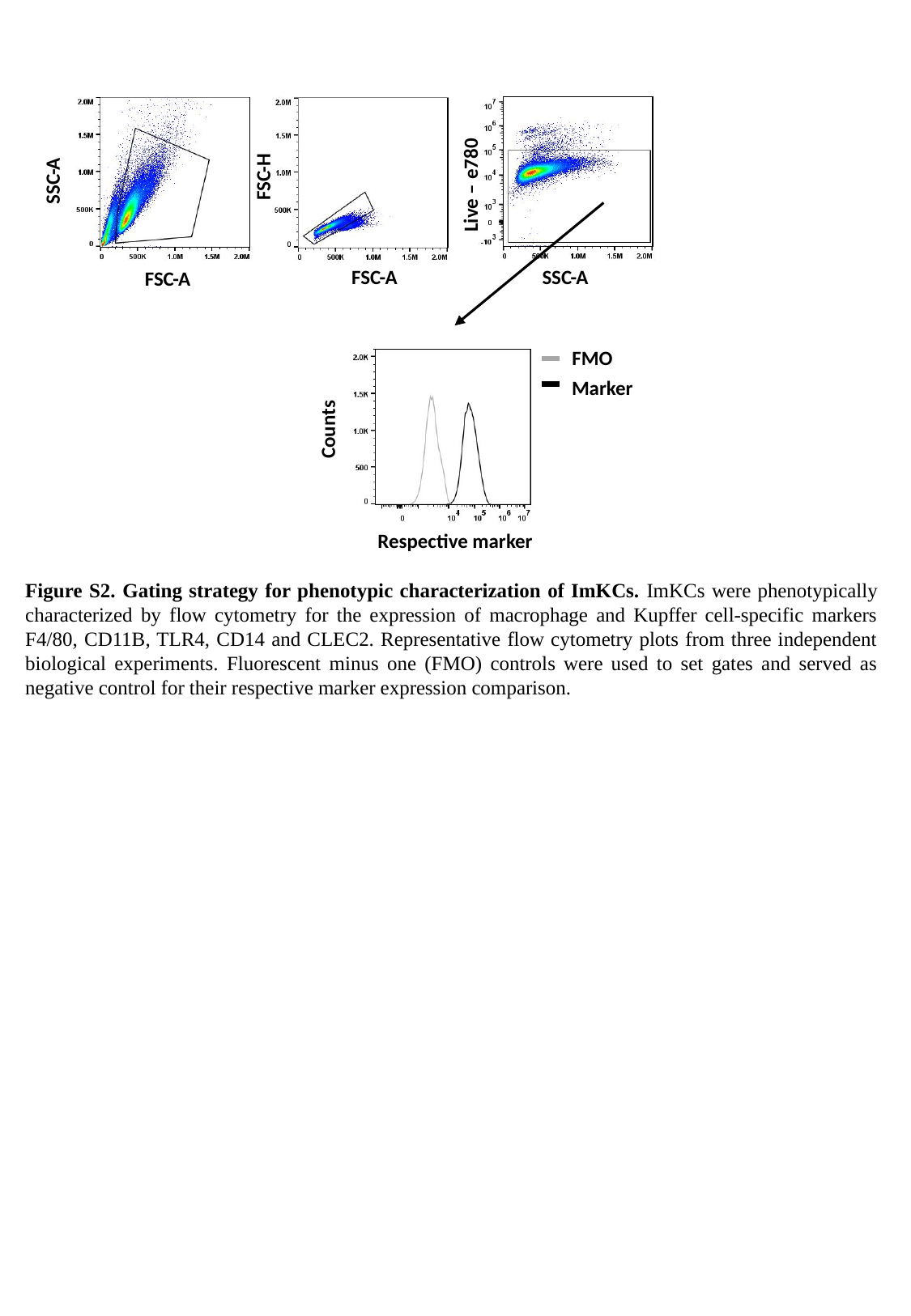

FSC-H
SSC-A
Live – e780
SSC-A
FSC-A
FSC-A
FMO
Marker
Counts
Respective marker
Figure S2. Gating strategy for phenotypic characterization of ImKCs. ImKCs were phenotypically characterized by flow cytometry for the expression of macrophage and Kupffer cell-specific markers F4/80, CD11B, TLR4, CD14 and CLEC2. Representative flow cytometry plots from three independent biological experiments. Fluorescent minus one (FMO) controls were used to set gates and served as negative control for their respective marker expression comparison.

## Slide 3
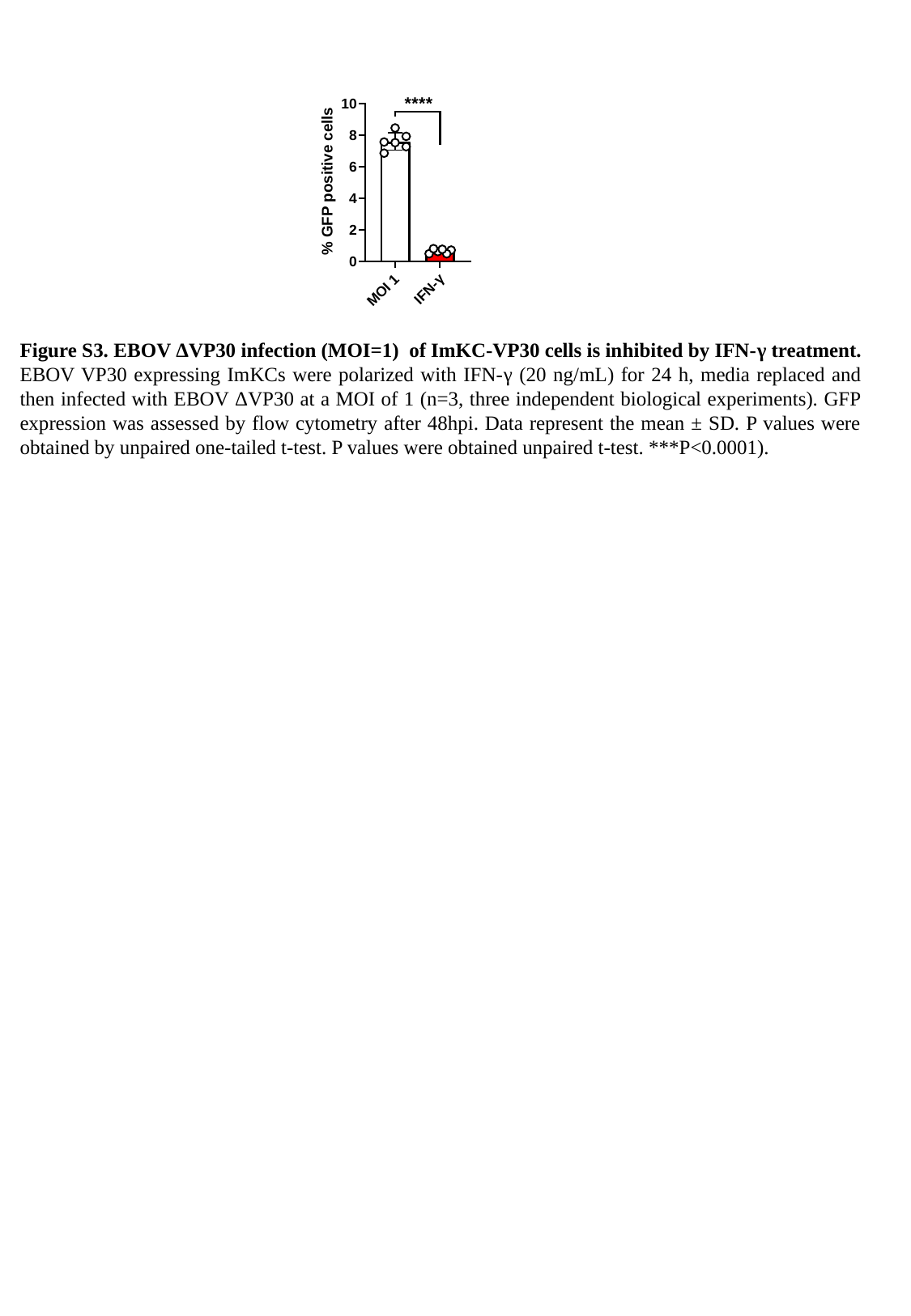

Figure S3. EBOV ΔVP30 infection (MOI=1) of ImKC-VP30 cells is inhibited by IFN-γ treatment. EBOV VP30 expressing ImKCs were polarized with IFN-γ (20 ng/mL) for 24 h, media replaced and then infected with EBOV ΔVP30 at a MOI of 1 (n=3, three independent biological experiments). GFP expression was assessed by flow cytometry after 48hpi. Data represent the mean ± SD. P values were obtained by unpaired one-tailed t-test. P values were obtained unpaired t-test. ***P<0.0001).

## Slide 4
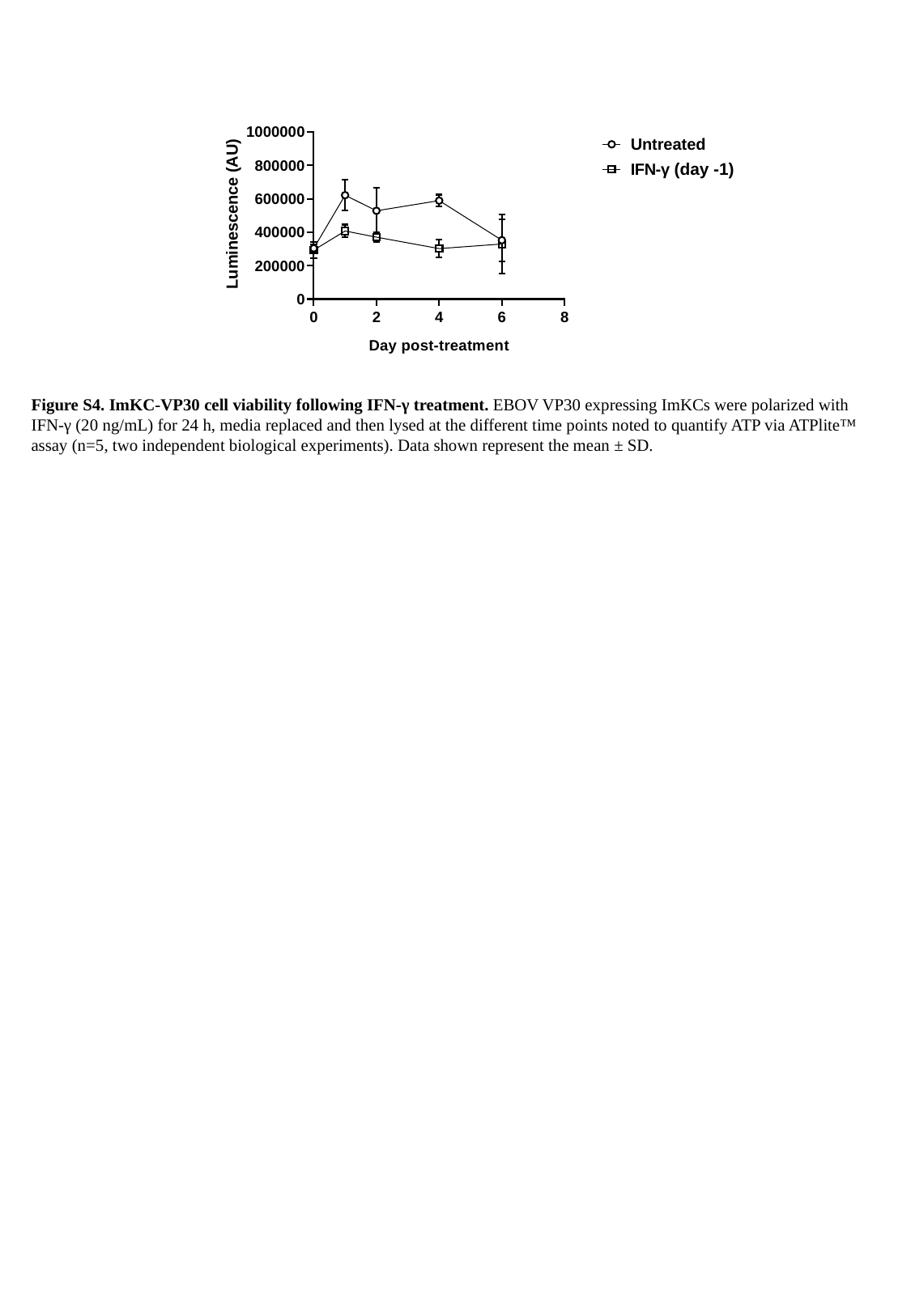

(day -1)
Figure S4. ImKC-VP30 cell viability following IFN-γ treatment. EBOV VP30 expressing ImKCs were polarized with IFN-γ (20 ng/mL) for 24 h, media replaced and then lysed at the different time points noted to quantify ATP via ATPlite™ assay (n=5, two independent biological experiments). Data shown represent the mean ± SD.
